# Retrograde Pulmonary Vein Recanalization Using Transcatheter Electrosurgery

**DOI:** 10.1016/j.jaccas.2022.03.026

**Published:** 2022-05-18

**Authors:** Saleem I. Almasarweh, James A. Kuo, Holly D. Bauser-Heaton, Vasilis C. Babaliaros, Dennis W. Kim

**Affiliations:** a Division of Cardiology, Children’s Healthcare of Atlanta, Atlanta, Georgia, USA; b Structural Heart and Valve Center, Emory University Hospital, Atlanta, Georgia, USA

**Keywords:** congenital heart disease, pediatric, pulmonary vein stenosis, transcatheter electrosurgery, LA, left atrium, LLPV, left lower pulmonary vein, LUPV, left upper pulmonary vein, RUPV, right upper pulmonary vein

## Abstract

Transcatheter electrosurgery is a wire-based technique used to traverse or cut tissue within blood-filled spaces using alternating current delivered by guidewires or catheters. The use of transcatheter electrosurgical techniques in the pediatric population has been limited. We are reporting the first case of retrograde pulmonary vein recanalization using transcatheter electrosurgery. (**Level of Difficulty: Advanced.**)

## Central Illustration

**Figure:**
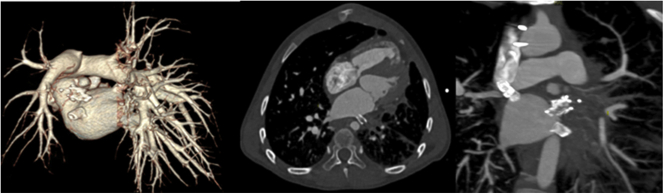

## History of Presentation

A 5-year-old, male patient with Trisomy 21 and prenatal diagnosis of Tetralogy of Fallot, initially underwent placement of a 4-mm right modified Blalock-Taussig shunt at 2 months of age. At 8 months of age, echocardiography showed evidence of pulmonary vein stenosis involving the left upper pulmonary vein (LUPV) and the left lower pulmonary vein (LLPV). Initial cardiac catheterization also demonstrated stenosis of the right upper pulmonary vein (RUPV). He underwent angioplasties as well as stenting of the RUPV and LLPV before his complete “valve-sparing” tetralogy of Fallot repair with “sutureless” repair of the left pulmonary veins and transection of the RUPV stent at 21 months of age. Despite this, he continued to have progressive pulmonary vein disease requiring repeated pulmonary vein recanalizations/angioplasties as well as repeat RUPV and LLPV stenting. The LUPV became atretic and could not be recanalized, leaving the LLPV as the only egress of pulmonary venous flow from the left lung.

**Learning Objectives**

• To highlight that tools and techniques for coronary chronic total occlusion can be adapted for pediatric vascular occlusion therapies.
• To understand the role of established electrosurgical techniques in adult interventions being used in severe cases of pulmonary vein stenosis.
• To demonstrate that retrograde recanalization of atretic pulmonary veins can be performed if collateral vessels exist.

## Investigations

**Figure 1:**
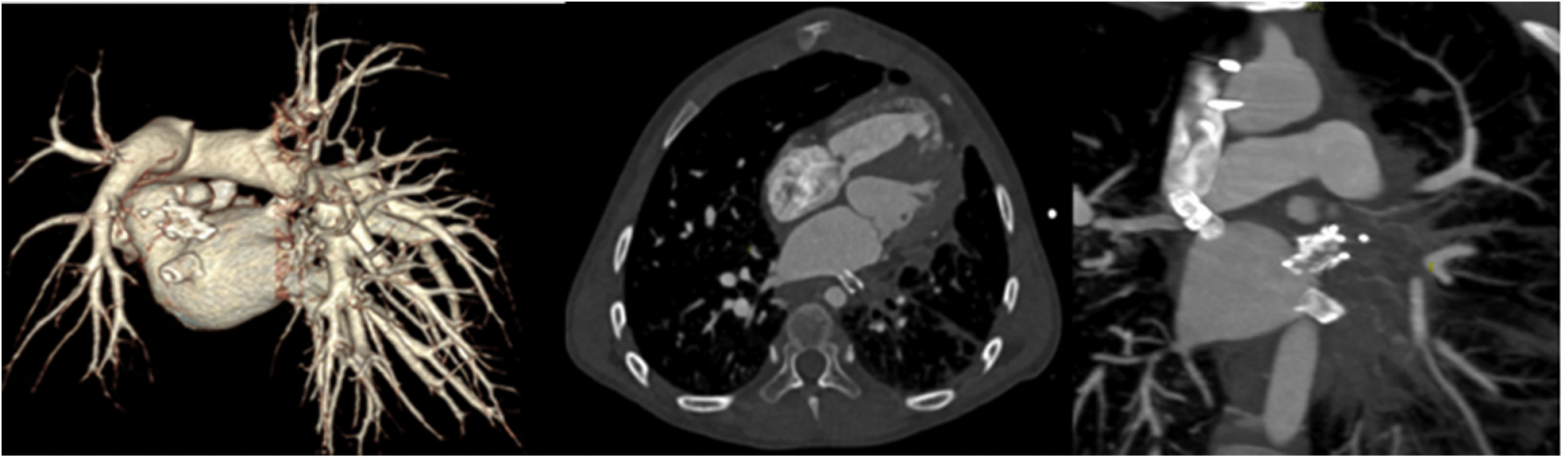
Computed Tomography Angiography Computed tomography angiography before intervention, demonstrating complete occlusion of left upper pulmonary vein and left lower pulmonary vein stents.

He was brought to the catheterization lab, after a follow-up computed tomography angiography demonstrated occlusion of the stented LUPV and LLPV (Figure 1).

## Management

**Figure 2:**
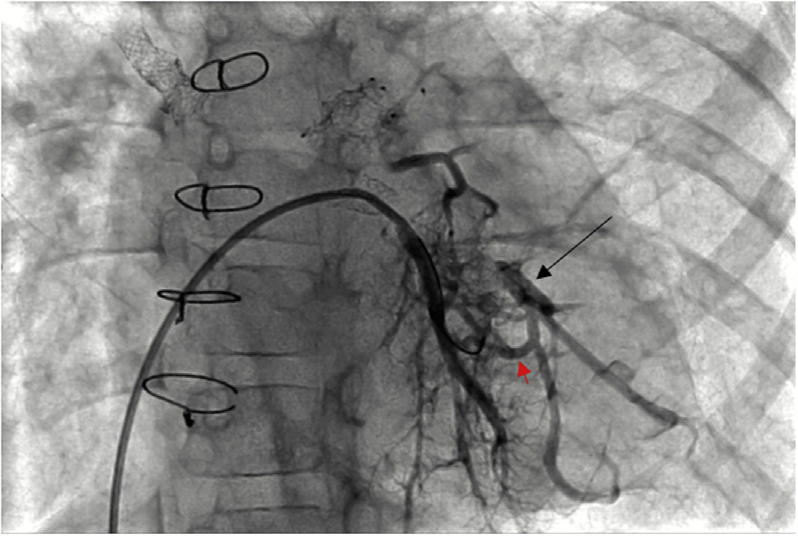
Angiogram Straight anteroposterior projection of hand injection in the left lower pulmonary vein after initial balloon angioplasty, demonstrating long segment atresia from the peripheral bifurcation point to the pulmonary vein stent of a posterior-lateral major segmental branch of left lower pulmonary vein branch **(arrow)** supplied by collaterals **(red arrowhead)** from the more medial branches.

**Figure 3:**
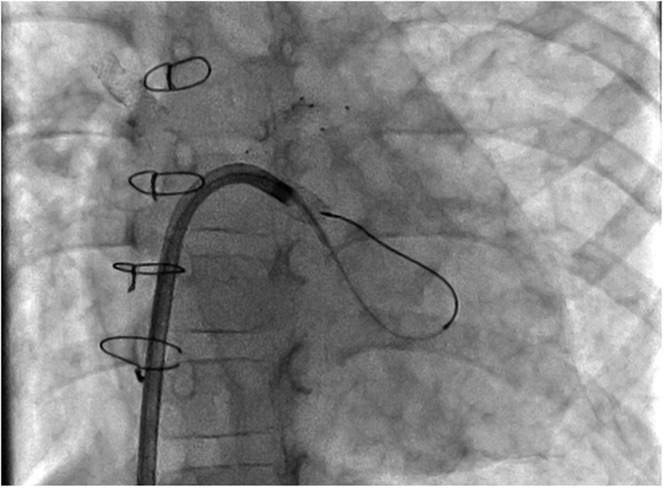
Microcatheter Position Straight anteroposterior projection, demonstrating the Echelon 10 microcatheter position after it was advanced retrograde through the venovenous collateral.

**Figure 4:**
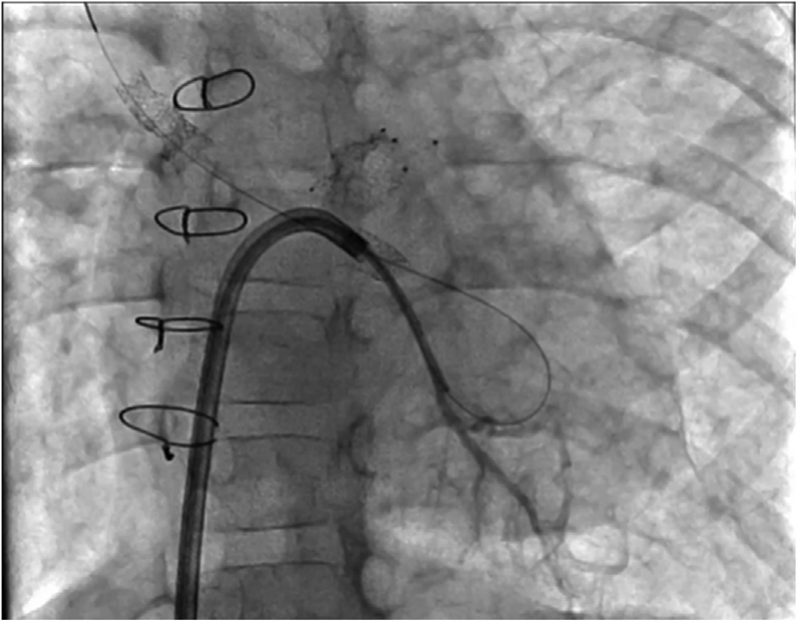
Electrosurgical Wire Position Straight anteroposterior (AP) projection, demonstrating the position of the balance middleweight wire in the right upper pulmonary vein after successful electrosurgical wire passage.

**Figure 5:**
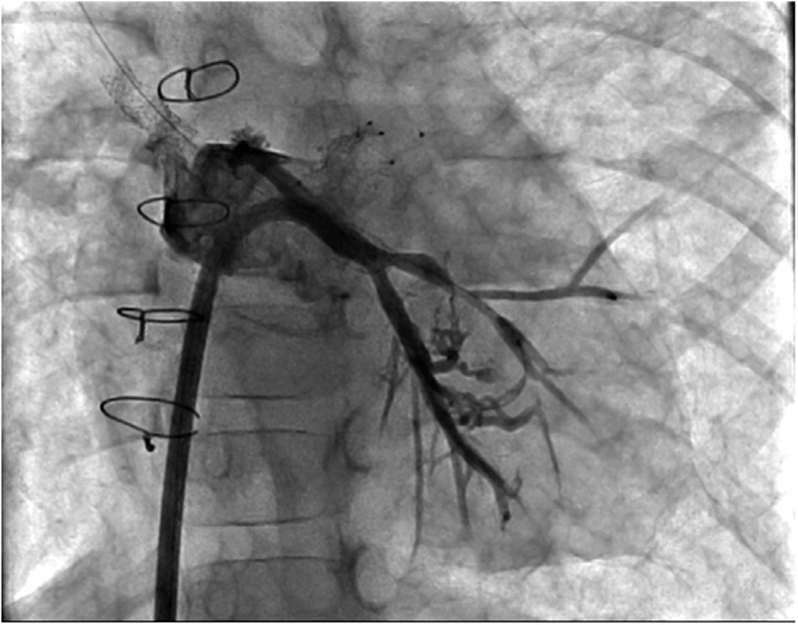
Final Result Straight anteroposterior projection of a hand injection in the left lower pulmonary vein, after recanalization of the atretic branch, demonstrating continuity into the left atrium.

Right femoral venous access was obtained using a 5-F sheath, after obtaining access to the left atrium (LA), a 6.5-F Destino Twist steerable guiding sheath (Oscor Inc.) was advanced to the LA. Angiography showed complete occlusion of the LLPV stent; however, a guidewire was able to be advanced to the distal left lower segmental branch (Video 1). After balloon angioplasty and improvement of flow, it was noticed that another posterior-lateral major segmental LLPV branch had a long segment atresia with discontinuity to the LLPV stent with the distal vessel supplied by collaterals from the more medial branches (Video 2, Figure 2). Multiple attempts that were made to obtain access to this branch from the LA through the LLPV stent were unsuccessful. Through a venovenous collateral vessel, a Hi-Torque Whisper wire (Abbott Inc.) was able to be passed retrograde back toward the LLPV stent over which an Echelon 10 microcatheter, a Nitinol braided microcatheter typically used in small vessel peripheral and neurovascular interventions (Medtronic), was then able to traverse through the venous collateral vessel into the major branch and back toward the stent more medially (Figure 3). However, retrograde reentry into the LLPV stent was not able to be obtained. Although the direction of blood flow is toward the LA from the peripheral segmental pulmonary veins, we use the term “retrograde” to indicate intervention directed back toward the pulmonary vein ostium to be consistent with this term being used for total occlusion coronary artery interventions that are performed via collateral vessels to reestablish flow to the occluded segment. Electrosurgical wire passage (Video 3) was performed by advancing the distal end of a straight tip Balance Middleweight coronary guidewire, protruding just beyond the microcatheter catheter tip with the backend of the guidewire clamped to the electrosurgery pencil with a hemostat, 50 W was delivered for <2 seconds using the electrosurgery pen and generator (Medtronic Valleylab FT10 Energy Platform). The guidewire easily was advanced across the tissue passing through the LLPV stent into the LA and the wire was able to be passed to the RUPV (Figure 4). Because the electrosurgery wire technique directs energy to such a small area in contact with the 0.014-inch-diameter wire, thermal exposure to other adjacent structures is avoided. Retrograde serial balloon angioplasties of the recanalized segmental pulmonary vein were performed using 1.5-mm and 3-mm noncompliant coronary angioplasty balloons (Video 4), followed by kissing balloon angioplasty of the LLPV stent into the 2 major peripheral branches using two 3-mm noncompliant coronary balloons (Video 5). After this, continuity to LA was established without evidence of vessel injury or contrast extravasation (Videos 6A and 6B, Figure 5).

## Follow-Up

Patient was discharged home the next morning with no clinical evidence of esophageal or phrenic nerve injury.

## Discussion

Pulmonary vein stenosis in children is a challenging disease, because of its progressive nature,^1^^,^^2^ high reintervention rates after percutaneous balloon and stent angioplasty, and poor long-term survival.^3^^,^^4^ Transcatheter electrosurgery is a technique used to traverse or cut tissue, within blood-filled spaces, using alternating current directed by guidewires and catheters.^5^ In contrast to adults, the use of transcatheter electrosurgical techniques in the pediatric population has been limited. The Baylis radiofrequency perforating system has been used to perforate atretic pulmonary valve plates in pulmonary atresia intact ventricular septum,^6^, ^7^, ^8^ and for atrial septal puncture in newborns with hypoplastic left heart syndrome and intact atrial septum,^8^, ^9^, ^10^ but requires a specialized system with a relatively large microcatheter needed. We report a novel procedure to recanalize atretic pulmonary veins via a retrograde approach and using electrosurgery to transverse atretic tissue for vessel recanalization in a pediatric patient.

## Funding Support and Author Disclosures

The authors have reported that they have no relationships relevant to the contents of this paper to disclose.

## Appendix

Supplemental Video 1 Initial Angiography Angiography demonstrating complete occlusion of the LLPV stent.

Supplemental Video 2 Angiography After Initial Balloon Angioplasty Angiography demonstrating long segment atresia of a posterior-lateral major segmental left lower pulmonary vein branch with the distal vessel supplied by collaterals from the more medial branches.

Supplemental Video 3 Electrosurgical Wire Passage Fluoroscopy demonstrating electrosurgical wire passage retrograde through the venous collateral into the LA.

Supplemental Video 4 Retrograde Balloon Angioplasty Fluoroscopy demonstrating retrograde balloon angioplasty using a 3-mm noncompliant coronary angioplasty balloon.

Supplemental Video 5 Kissing Balloon Angioplasty Fluoroscopy demonstrating kissing balloon angioplasty of the LLPV stent into the 2 major peripheral branches using two 3-mm noncompliant coronary balloons.

Supplemental Video 6 Final Result **(A and B)** Straight AP and lateral projection of a hand injection demonstrating continuity to LA at the end of the procedure.

Supplemental Video 6 Final Result **(A and B)** Straight AP and lateral projection of a hand injection demonstrating continuity to LA at the end of the procedure.7
